# Formation of Monodisperse Carbon Spheres with Tunable Size via Triblock Copolymer-Assisted Synthesis and Their Capacitor Properties

**DOI:** 10.1186/s11671-019-2952-8

**Published:** 2019-04-03

**Authors:** Zhongguan Liang, Luomeng Zhang, Hao Liu, Jianping Zeng, Jianfei Zhou, Hongjian Li, Hui Xia

**Affiliations:** 1 School of Physics and Electronics, Central South University, Changsha, 410083 China; 2 School of Physics and Electronics, Hunan University, Changsha, 410082 China

**Keywords:** Surfactant, Triblock Copolymer F108, Monodisperse carbon spheres, Supercapacitor, Energy storage and conversion, 81.05.Uw, 88.80.Fh, 82.47.Uv

## Abstract

A facile hydrothermal polymerization method has been developed for the preparation of monodisperse carbon spheres (MCSs) using the triblock copolymer F108 as surfactant. The synthesis is based on the ammonia-catalyzed polymerization reaction between phenol and formaldehyde (PF). The resultant MCSs have a perfect spherical morphology, smooth surface, and high dispersity. The particle sizes can be tuned in a wide range of 500~2400 nm by adjusting the dosage of the PF precursor. The activated MCSs with suitable heteroatoms (N and O) doped and a large specific surface area (960 m^2^ g−^1^) were obtained. A high-performance electrode of electrical double-layer capacitors fabricated by those active material have an excellent specific capacitance (310 F g^−1^ at 0.5 A g^−1^) and outstanding cycling stability (92% capacitance retention after 10,000 cycles). This work provides a new opportunity for the fabrication of MCSs with potential applications.

**Electronic supplementary material:**

The online version of this article (10.1186/s11671-019-2952-8) contains supplementary material, which is available to authorized users.

## Introduction

Over the past few decades, porous carbon materials have been widely used in the fields of gas storage [1], catalyst supports [2], supercapacitors [3], lithium-ion batteries [4], solar cells [5], and electronic devices [6] due to their advantages like the high-specific surface area, good electrical conductivity, and high chemical stability. From the perspective of materials chemistry, porous carbon materials with different morphology and structure such as carbon aerogels [7], fibers [8], nanotubes [9], nanospheres [10], and activated carbon [11] have been synthesized successfully. Recently, monodisperse carbon spheres (MCSs) have gained considerable investigation into functional electrode materials for energy storage and conversion devices because of the unique properties such as high stack density, inherent short ion diffusion path way, and good structural stability [12, 13]. The precise control over the morphology, dispersity, smooth surface, and particle size of the MCSs has been the key to meet the requirement for some special practical applications [14].

The carbonization of pre-synthesized phenolic resin polymer spheres with excellent thermal stability has been demonstrated to be a favored approach for the preparation of MCSs. Zhao group reported a low-concentration hydrothermal route to synthesize highly uniform ordered mesoporous carbon spheres with a tunable size from 20 to 140 nm by using phenolic resol as the carbon precursor [15]. By smartly associating the hydrolysis polymerization reaction mechanism of resorcinol-formaldehyde resins with the classical Stöber silica spheres, Liu and co-researchers successfully developed an extension of the Stöber method for the synthesis of MCSs with a uniform and controllable size on the submicrometer scale [16]. Based on benzoxazine chemistry, Lu and co-workers established a new way to synthesize high dispersity MCSs with tailored sizes in the range of 95~225 nm under precisely programmed reaction temperatures [17]. After these groundbreaking works, tremendous attention has been paid to the design and synthesis of MCSs [18–21]. However, most of those approaches either require tedious hydrothermal treatment processes, or cannot prepare a wide tunable particle size with smooth surface and narrow size distribution. Therefore, the synthesis of wide tunable sized, highly uniform, and morphological clearly defined MCSs still remains a grand challenge.

In this work, we propose a facile hydrothermal method for the preparation of MCSs using triblock copolymer Pluronic F108 as a surfactant based on the ammonia-catalyzed polymerization reaction of phenol and formaldehyde (PF). The detailed formation mechanism of MCSs has been discussed. The as-prepared MCSs have a perfect spherical morphology and smooth surface and are highly uniform. The particle sizes of MCSs can be tuned in a wide range of 500~2400 nm depending on the concentration of the PF precursor. When used as electrode materials for supercapacitors, the activated MCSs exhibit an excellent electrochemical performance due to the nitrogen and oxygen co-doping and high specific surface.

## Methods

### Synthesis of MCSs

In a typical synthesis, 0.5 mL ammonia aqueous (25 wt%) was mixed with 30 mL ethanol and 50 mL deionized water (H_2_O). Then, 10 mg Triblock copolymer Pluronic F108 (Mw = 14,600, PEO_132_-PPO_50_-PEO_132_) was dissolved in the mixture solution. Next, 0.2 mL phenol and 0.2 mL formaldehyde (37 wt%) were added respectively, with gentle stirring for 30 min. Finally, the resulting solution was transferred to a 100-mL Teflon-lined autoclave and hydrothermal reaction was regulated at 160 °C for 3 h. The resulting PF resin polymer spheres were obtained by washing with H_2_O and ethanol for several times. Then, MCSs-x were obtained by annealing the PF resin spheres under N_2_ atmosphere at 600 °C for 3 h, “x” denotes the phenol and formaldehyde dosage used (for example 0.2, 0.4, 0.6, and 0.8 refer to 0.2, 0.4 ,0.6, and 0.8 mL of phenol and formaldehyde, respectively). The MCSs-x were further chemically activated by KOH (in a mass ratio of 1:2) at 700 °C for 1 h in a N_2_ atmosphere to prepare the aMCSs-x.

### Characterization

Scanning electron microscopy (SEM) was performed on a NovaNanoSEM230 instrument. Transmission electron microscopy (TEM) was conducted on a Tecnai G2 F20 S-TWIX instrument. X-ray diffraction (XRD) patterns were carried out with a SIEMENS D500 diffractometer with Cu Kα radiation (*λ* = 0.15056 nm). Raman spectroscopy was performed on a LabRAMHR-800 system. X-ray photo-electron spectroscopy (XPS) analysis was conducted on an ESCALAB 250Xi instrument. Nitrogen adsorption-desorption isotherms were measured at 77 K on an ASAP 2020 instrument.

### Electrochemical Measurement

The electrochemical test of cyclic voltammetry (CV), galvanostatic charge/discharge (GCD), and electrochemical impedance spectroscopy (EIS) was conducted on the CHI660E electrochemical workstation with a three-electrode system in 6 M KOH electrolyte solution. The platinum foil and Hg/HgO were used as the counter electrode and reference electrode, respectively. The working electrodes were fabricated by the mixing of the aMCSs-x, polytetrafluoroethylene (60 wt%), and acetylene black with a mass proportion of 8:1:1. The gravimetric-specific capacitance was calculated by the following equation:

1 $$ Cg=\frac{I\Delta t}{m\Delta V} $$

where *I* (A), Δ*t* (s), Δ*V* (V), and *m* (g) are the applied current, discharge time, potential window, and the active material mass of the electrodes, respectively.

## Results and Discussion

**Scheme 1:**
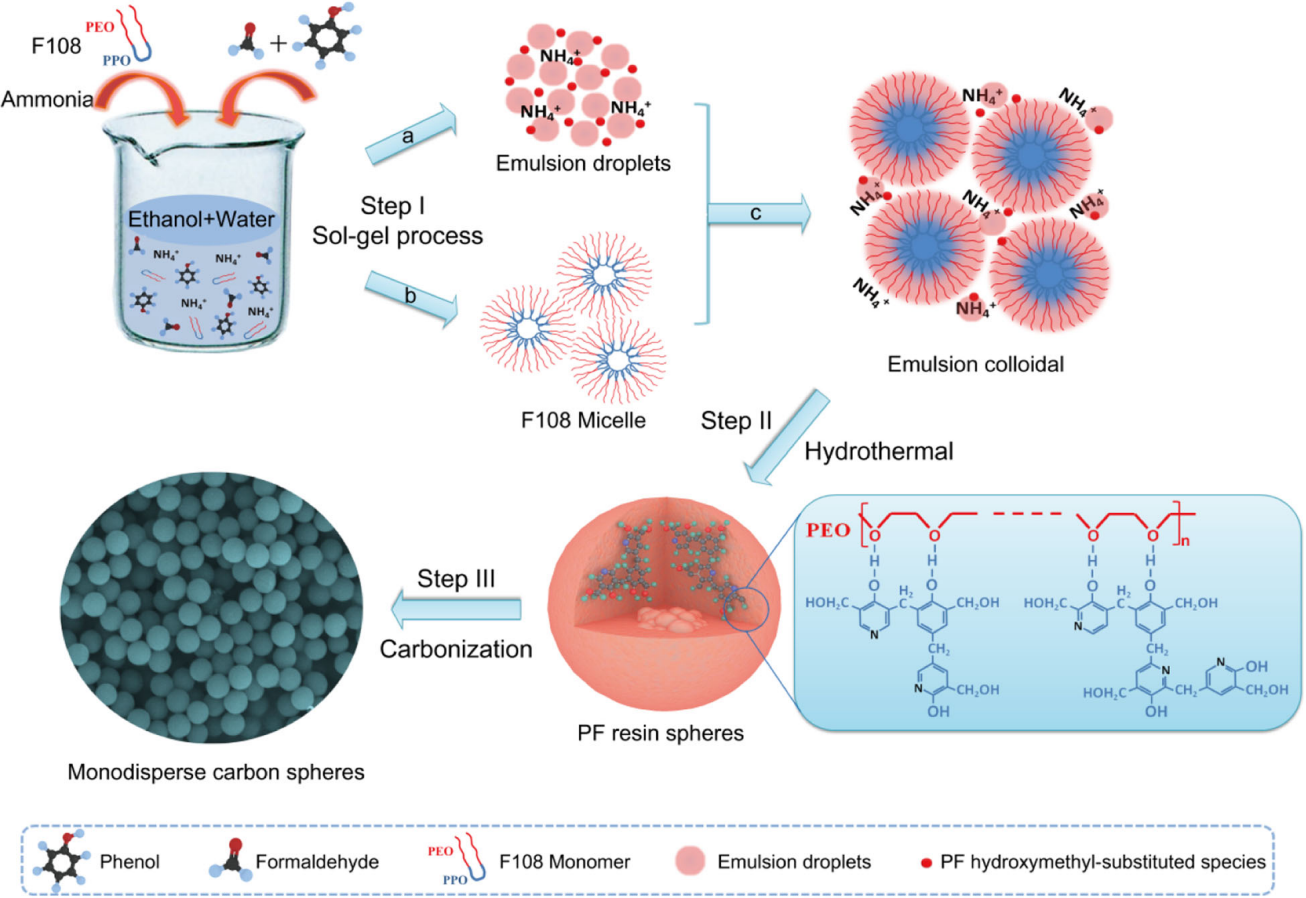
The synthesis process of MCSs

In this study, we present a possible synthesis mechanism of the MCSs in Scheme 1. Step I is the sol-gel process. In path a, the emulsion droplets formed through the hydrogen-bonding interaction between phenol, formaldehyde, ammonia molecule, ethanol, and water [16]. Ammonia molecules catalyze the polymerization of PF which takes place from the inside of the emulsion droplets [22]. In addition, a large number of PF hydroxymethyl substituted units are produced by rapid reaction of phenol and formaldehyde, which are positioned at the outer surface of the emulsion droplets because of the electrostatic interaction with ammonia ions. Simultaneously, path b shows the self-forming process of F108 micelles formed by triblock copolymer F108 monomers, which are the hydrophobic PPO blocks to form the nucleus inside and the hydrophilic PEO segments outside [23]. Then, in path c, abundant emulsion droplets/PF hydroxymethyl-substituted species can interact with the hydrophilic PEO segments of F108 micelles via hydrogen-bonding interaction to form the emulsion colloidal [24]. In step II, under mild hydrothermal treatment conditions, the species are for further cross-linking polymerization and result in uniform PF resin/F108 copolymer spheres. Finally, in step III, the PF resin/F108 copolymer spheres are followed by carbonization at a high temperature to obtain the MCSs.

**Fig. 1:**
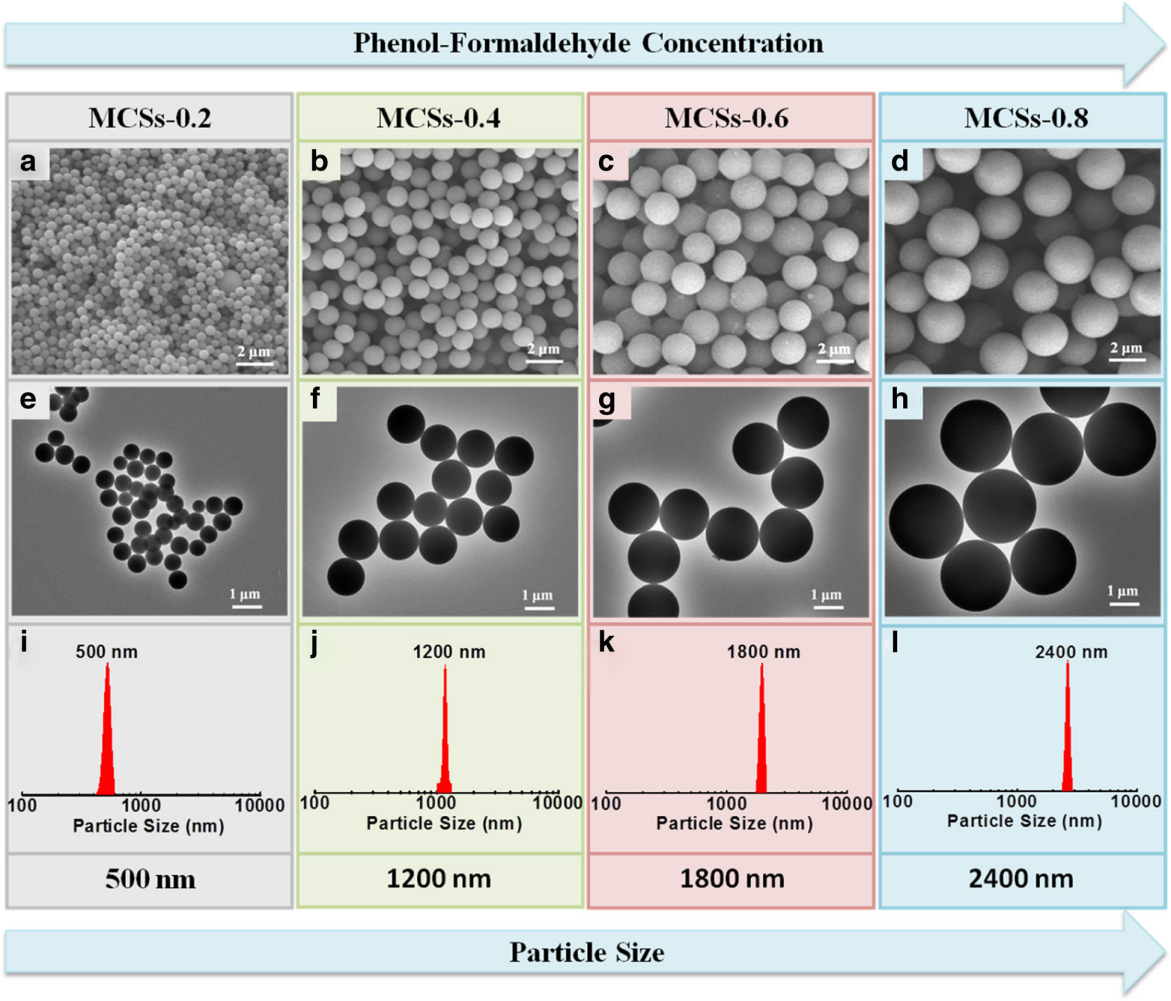
SEM and TEM images of MCSs prepared at different phenol and formaldehyde dosages of **a**, **e** 0.2 mL, **b**, **f** 0.4 mL, **c**, **g** 0.6 mL, and **d**, **h** 0.8 mL, respectively. **i**–**l** The particle size distribution of MCSs corresponding to the SEM images (**a**–**d**)

The SEM images of MCSs prepared at different PF dosages shown in Fig. 1a–d demonstrate that the MCSs have a perfect spherical morphology with a uniform size. TEM images present in Fig. 1e–h further confirm the MCSs have spherical particles, smooth surface, and high dispersity. The average particle diameter increased from 500 to 2400 nm with the increasing dosage of the PF precursor from 0.2 to 0.8 mL, as shown in Fig. 1i–l. This is because the increasing concentration of the PF precursor led to emulsion droplets and colloidal with a larger size and resulted in a larger final MCS diameter. The use of ammonia in this system is critical to the successful synthesis of such highly dispersive MCSs, which can provide the NH_4_^+^ to adhere to the surface of PF spheres and inhibit the aggregation. It is noticed that the MCSs were without any obvious surface defect and structure collapse after high-temperature carbonization. This is the main benefit from the high cross-linking reaction between phenol and formaldehyde. In addition, we also investigated the role of triblock copolymer F108 in this system. Additional file 1: Figure S1a presents the SEM image of the carbon spheres obtained in the absence of F108. The products have a non-uniform particle size and encounter agglomeration. Furthermore, the particle size decreases systematically with increasing F108 dosage from 20 to 80 mg, and small particles and flaky substances appear on the surface of carbon spheres and finally encounter great conglutination (Additional file 1: Figure S1b~d). The reason is that when the triblock copolymer F108 is sufficient in the system, the surface tension decreases, stronger cross-linking interaction occurs, and smaller-sized emulsion droplets and carbon spheres are formed. However, appropriate F108 concentration can balance the surface tension and cross-linking interaction forces and obtain the smooth surface and uniformly sized carbon spheres. In addition, the effect of F108 concentration on the electrode properties of the MCSs was also investigated, as shown in Additional file 1: Figure S2. The result reveals that the triblock copolymer F108 played as a surface active agent for the formation of MCSs.

**Fig. 2:**
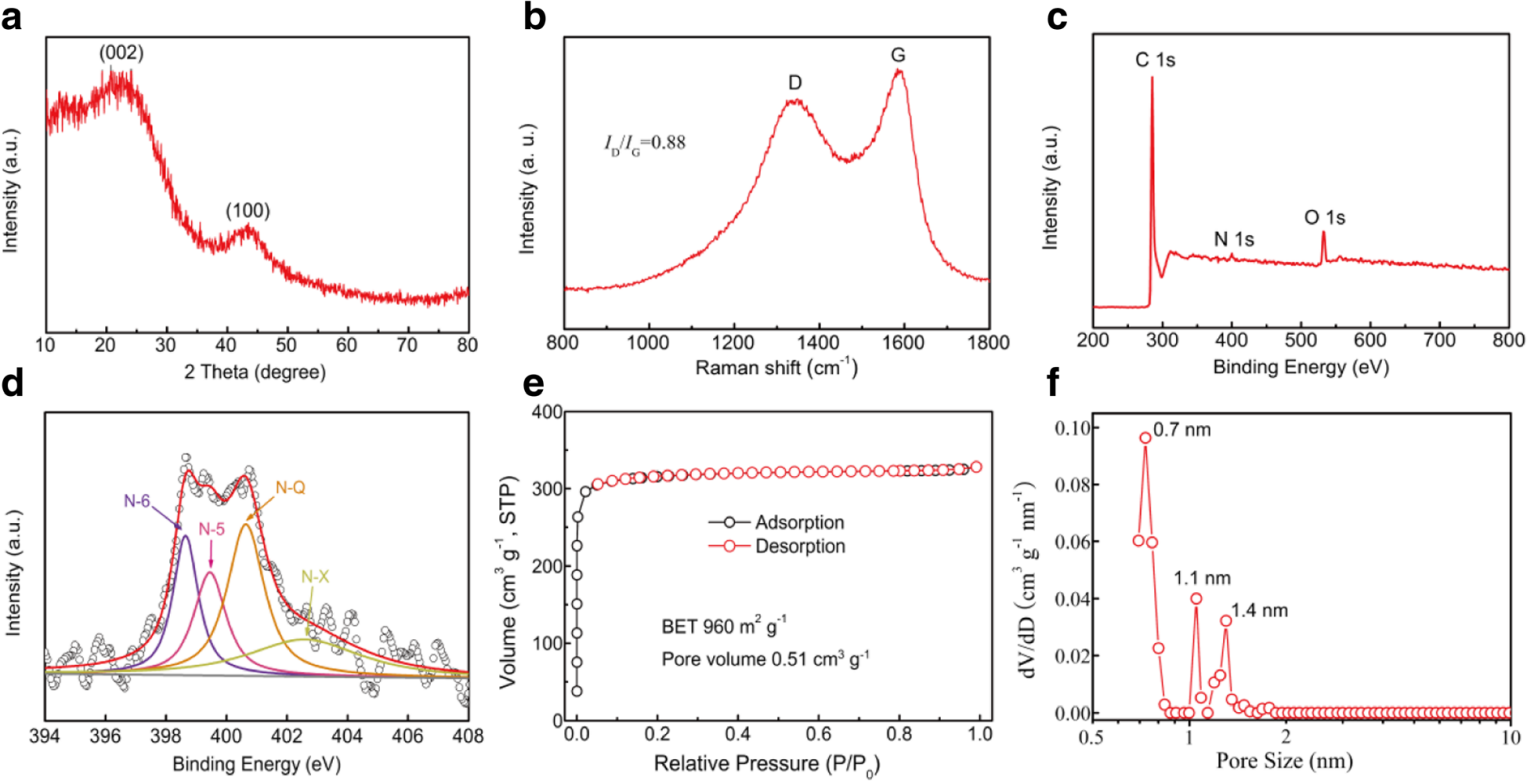
**a** XRD pattern. **b** Raman spectra. **c** XPS survey spectrum. **d** High-resolution N 1s spectra. **e** Nitrogen adsorption/desorption isotherms. **f** Pore size distribution curve of the aMCSs-0.4 material

These synthesized MCSs may have some potential applications such as catalysis, adsorption, and electrode materials for supercapacitors and lithium-ion batteries. In order to understand the structure property of the as-prepared material, the aMCSs-0.4 was selected as a sample further used for characterization analysis. As shown in Fig. 2a, the XRD pattern of aMCSs-0.4 displays two obvious broad diffraction peaks at 25° and 43°, corresponding to the (002) and (100) lattice planes of the amorphous carbon material, respectively. It also indicates the PF resin’s complete conversion to carbon material and the almost removal of triblock copolymer F108 after carbonization. The Raman spectrum of the aMCSs-0.4 (Fig. 2b) exhibits two typical peaks at 1337 cm^−1^ (D band) and 1590 cm^−1^ (G band), which correspond to the crystal defects and the hexagonal graphitic property of carbon materials, respectively. The intensity ratio (*I*_D_/*I*_G_) of carbon materials reflects the graphitization degree [25]. The *I*_D_/*I*_G_ value of the aMCSs-0.4 is about 0.88, which also corroborates the amorphous structures.

As shown in Fig. 2c, the XPS survey of the aMCSs-0.4 exhibits three peaks of C 1s (285.2 eV), N 1s (400.1 eV), and O 1s (532.7 eV). The elemental compositions of C, N, and O in aMCSs-0.4 are 92.54 at%, 1.04 at%, and 6.42 at%, respectively. The results suggest that the ammonia can act as a source of nitrogen to introduce the N element into the carbon frameworks. Figure 2d displays the high-resolution N 1s spectrum of aMCSs-0.4. Four type peaks at 398.6 eV, 399.4 eV, 400.6 eV, and 402.4 eV are correlated to pyridinic-N (N-6), pyrrolic-N (N-5), quaternary-N (N-Q), and pyridine-N-oxides (N-X), respectively [10]. Generally, the presence of nitrogen-based functional groups can not only contribute to addition pseudocapacitance but also can improve the surface wettability and electric conductivity of carbon materials, and thus enhance electrochemical performance [3, 26].

The N_2_ adsorption/desorption measurements were conducted to investigate the specific surface areas and internal pore structure of the prepared materials. As shown in Fig. 2e, the isotherm of aMCSs-0.4 belongs to a typical type I curve with a steep uptake at low relative pressures, and an almost horizontal plateau at higher relative pressures reveals the microporous structure. The BET surface areas and total pore volume of aMCSs-0.4 are determined to be 960 m^2^ g^−1^ and 0.51 m^3^ g^−1^, respectively. The pore size distribution curve of aMCSs-0.4 is shown in Fig. 2f, which exhibits the micropore structure with diameters of 0.7 nm, 1.1 nm, and 1.4 nm. The high-resolution TEM image (Additional file 1: Figure S3) is also in good agreement with this result. The micropore carbon structures are generated from the decomposition of F108 during carbonization and the chemical activity of KOH [27, 28].

**Fig. 3:**
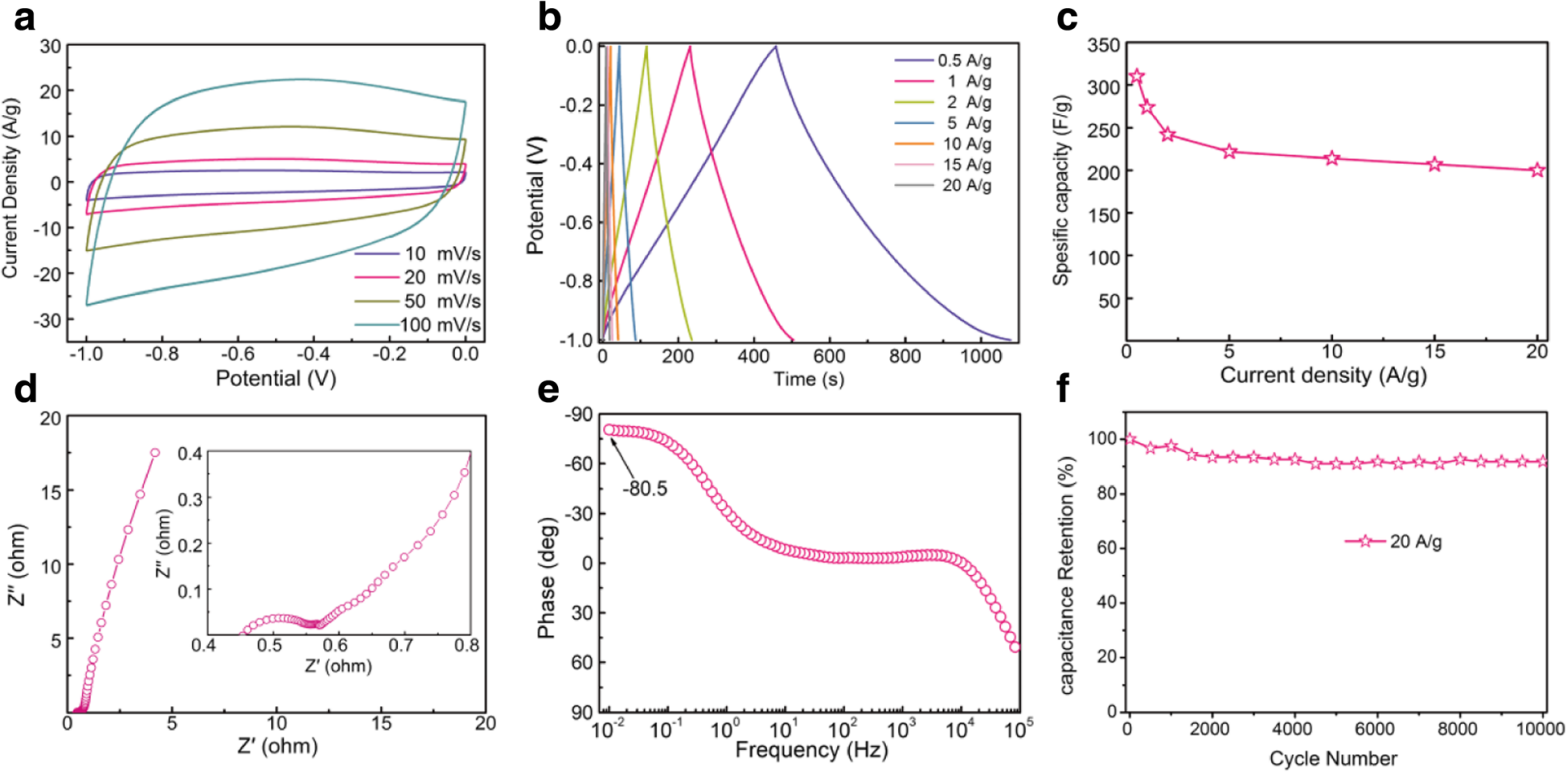
The electrochemical characteristics of the aMCSs-0.4 electrode. **a** CV curves at different scan rates from 10 to 100 mV s^−1^. **b** GCD curves at different current densities from 0.5 to 20 A g^−1^. **c** Specific capacitance as a function of current densities. **d** Nyquist plot and the inset show the magnify plots at a high-frequency range. **e** Bode plot. **f** Cycling performance at a current density of 20 A g^−1^ for 10,000 cycles

Here, we employ the aMCSs-0.4 as electrode materials for electrical double-layer capacitors (EDLCs) to demonstrate their structural and performance advantages. The CV curves of aMCSs-0.4 electrode exhibit rectangular shapes at different scan rates from 10 to 100 mV s^−1^ (Fig. 3a), and the GCD curves display typical triangular profiles (Fig. 3b). These reveal that the aMCSs-0.4 materials have a perfect EDLC performance. As shown in Fig. 3c, the aMCSs-0.4 electrode exhibits an excellent specific capacitance of 310 F g^−1^ at a current density of 0.5 A g^−1^, which is higher than other similar MCS electrodes [12–14]. The high specific capacitance benefits from the large surface areas and heteroatoms doped. In addition, the specific capacitance still maintains 200 F g^−1^ even at a large current density of 20 A g^−1^; it exhibits good capacitance retention. The charge transport and transfer kinetic behaviors can be examined by EIS. The Nyquist plot of the aMCSs-0.4 electrode (Fig. 3d) features a small internal resistance (0.45 Ω) and charge transfer resistance (0.12 Ω) revealing the high electron conductivity of the prepared aMCSs-0.4 materials and good electrode/electrolyte contact interface. The nearly vertical line in the low-frequency region suggests the aMCSs-0.4 electrode has an ideal capacitor property and efficient electrolyte ion diffusion. This result was further confirmed by Bode plots (Fig. 3e), which display the phase angle (− 80.5°) close to − 90°. Furthermore, the aMCSs-0.4 electrode shows good cycling stability with 92% retention over 10,000 cycles at a current density of 20 A g^−1^ (Fig. 3f). Therefore, from above, all results clearly highlight the attractive potential applications of MCSs for electrodes of EDLCs.

## Conclusions

In summary, we have demonstrated a facile surfactant-assisted hydrothermal method to effectively synthesize MCSs. The prepared MCSs have a perfect spherical morphology, uniform size, smooth surface, and tunable particle sizes in a wide range of 500~2400 nm. In particular, this methodology allows the aMCSs-0.4 to have unique structural features with a high surface area (960 m^2^ g^−1^) and suitable surface functionality of N and O co-doped. A high-performance electrode of EDLCs has been fabricated by using the aMCSs-0.4 as the active material which delivered an excellent specific capacitance (310 F g^−1^ at 0.5 A g^−1^) and outstanding cycling stability (92% capacitance retention after 10,000 cycles). This research provides a new opportunity for the fabrication of MCSs with potential applications.

## Additional file

Additional file 1: **Figure S1.** SEM images of MCSs prepared at different dosage of F108: (a) 0 mg, (b) 20 mg, (c) 40 mg and (d) 80 mg. **Figure S2.** The electrochemical characteristics of the aMCSs-0.4 electrode: (a) GCD curves with current density of 1 A/g at different F108 dosage, (b) Specific capacitance as a function of F108 dosage. **Figure S3.** The high-resolution TEM image of aMCSs-0.4. (DOCX 1965 kb)

## Abbreviations

CV: Cyclic voltammetry
EDLCs: Electrical double-layer capacitors
EIS: Electrochemical impedance spectroscopy
GCD: Galvanostatic charge/discharge
MCSs: Monodisperse carbon spheres
PF: Phenol and formaldehyde
