# Supplementary material for: Formation of Monodisperse Carbon Spheres with Tunable Size via Triblock Copolymer-Assisted Synthesis and Their Capacitor Properties
Source: Nanoscale Res Lett. 2019 Apr 3;14:124. doi: 10.1186/s11671-019-2952-8 (PMC6447644; doi:10.1186/s11671-019-2952-8)
Supplement: Supplementary file 1 — Figure S1. SEM images of MCSs prepared at different dosage of F108: (a) 0 mg, (b) 20 mg, (c) 40 mg and (d) 80 mg. Figure S2. The electrochemical characteristics of the aMCSs-0.4 electrode: (a) GCD curves with current density of 1 A/g at different F108 dosage, (b) Specific capacitance as a function of F108 dosage. Figure S3. The high-resolution TEM image of aMCSs-0.4. (DOCX 1965 kb) [file 11671_2019_2952_MOESM1_ESM.docx]

### Supplementary Information

**Formation of Monodisperse Carbon Spheres with Tunable Size via** [**Triblock Copolymer Assisted Synthesis**](https://pubs.acs.org/doi/abs/10.1021/ja029353j) **and their Capacitor Properties**

Zhongguan Liang^1^, Luomeng Zhang^1^, Hao Liu^1^, Jianping Zeng^2*^, Jianfei Zhou^1^, Hongjian Li^1^ and Hui Xia^1^*

*^1^**School of Physics and Electronics, Central South University, Changsha 410083, China.*

*^2^School of Physics and Electronics, Hunan University, Changsha 410082, China.*

*Corresponding author. Zengjp@hnu.edu.cn (J. Zeng), [xhui73@csu.edu.cn](mailto:xhui73@csu.edu.cn) (H. Xia).

PACS: 81.05.Uw; 88.80.Fh; 82.47.Uv


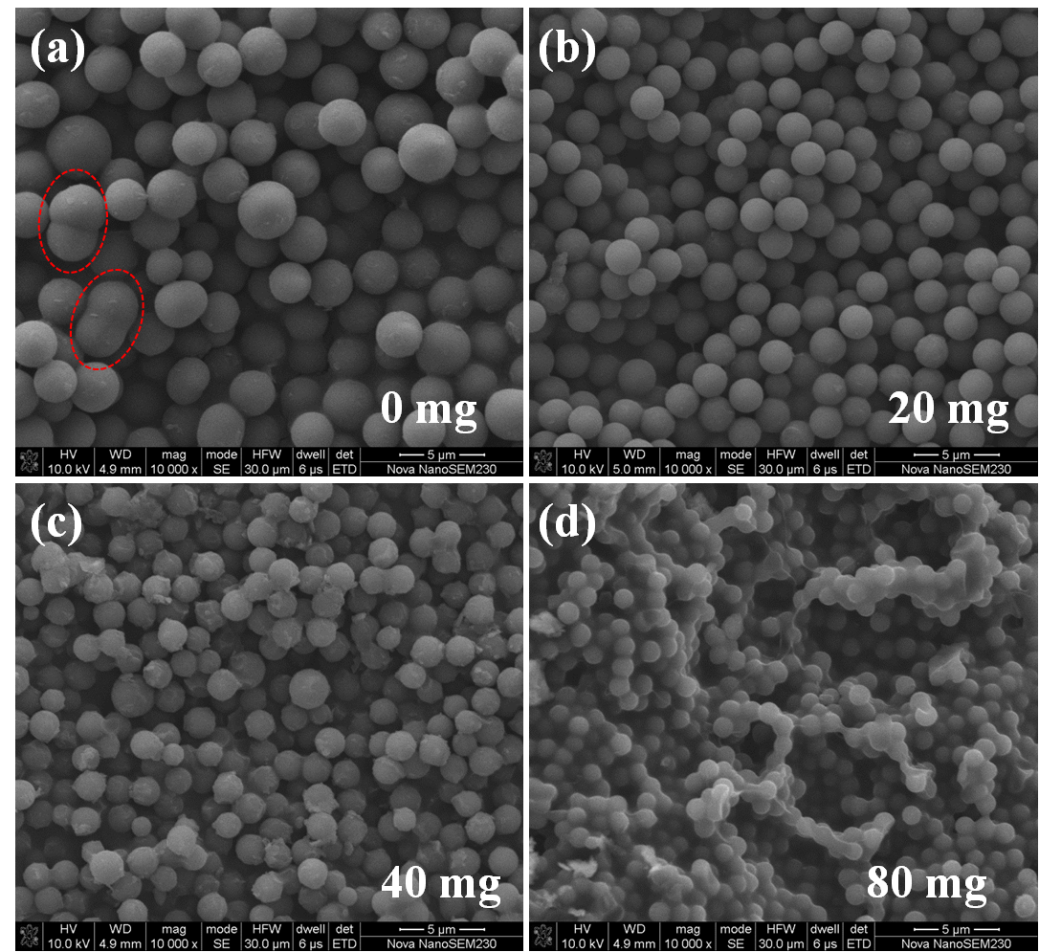
``

**Fig. S1** SEM images of MCSs prepared at different dosage of F108: (a) 0 mg, (b) 20 mg, (c) 40 mg and (d) 80 mg.


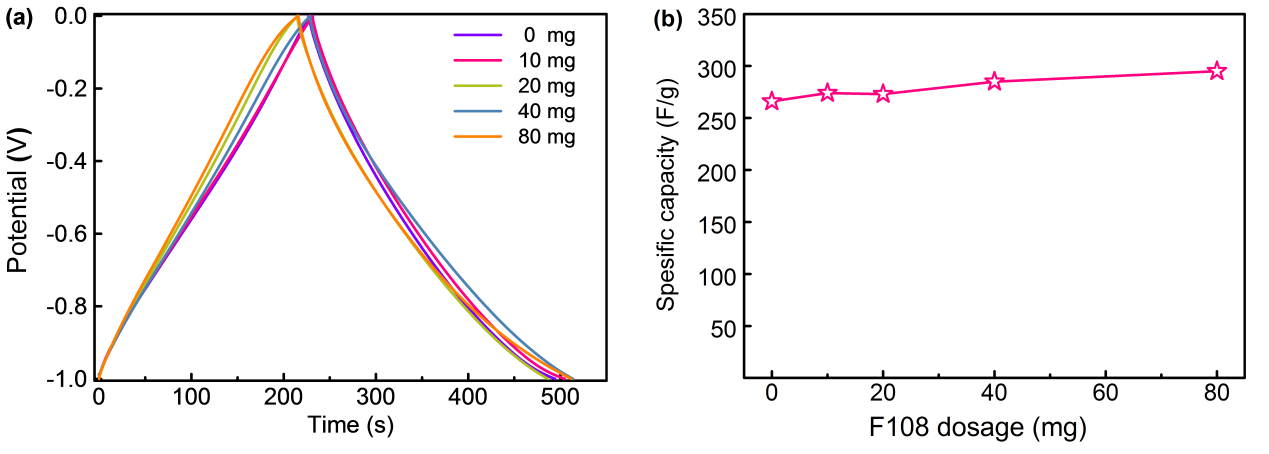


**Fig. S2** The electrochemical characteristics of the aMCSs-0.4 electrode: (a) GCD curves with current density of 1 A/g at different F108 dosage, (b) Specific capacitance as a function of F108 dosage.

As shown in Fig. S2 (a) and (b), the specific capacitance of aMCSs-0.4 electrode are slight increasing (from 266 A/g to 295 A/g) with the F108 dosage (from 0 mg to 80 mg). The resultant aMCSs-0.4 electrodes exhibit a size-dependent electrical double-layer capacitor performance, that is, the capacitance increases with decreasing particle size [1].

**

**

**Fig. S3** The high-resolution TEM image of aMCSs-0.4.

**References**

[1] Yu Q, Guan D, Zhuang Z, Li J, Shi C, Luo W, Zhou L, Zhao D, Mai L (2017) Mass Production of Monodisperse Carbon Microspheres with Size‐Dependent Supercapacitor Performance via Aqueous Self‐Catalyzed Polymerization. ChemPlusChem 82(6): 872-878
